# Correction to: Two sides of every coin: individuals’ experiences of undergoing colorectal cancer screening by faecal immunochemical test and colonoscopy

**DOI:** 10.1093/eurpub/ckac024

**Published:** 2022-03-11

*European Journal of Public Health*, 2021; https://doi.org/10.1093/eurpub/ckab171

In the originally published version of this manuscript, author forenames and surnames were swapped in error. These errors have been corrected online.

